# Supplementary material for: Origami: Single-cell 3D shape dynamics oriented along the apico-basal axis of folding epithelia from fluorescence microscopy data
Source: PLoS Comput Biol. 2021 Nov 1;17(11):e1009063. doi: 10.1371/journal.pcbi.1009063 (PMC8584784; doi:10.1371/journal.pcbi.1009063)
Supplement: S1 Text — (DOCX) [file pcbi.1009063.s002.docx]

Supplementary Materials and Methods

Origami: Single-cell 3D shape dynamics oriented along the apico-basal axis of folding epithelia from fluorescence microscopy data

Tania Mendonca, Ana A. Jones, Jose M. Pozo, Sarah Baxendale, Tanya T. Whitfield and Alejandro F. Frangi

*Zebrafish husbandry*

All zebrafish work was reviewed and approved by the Project Applications and Amendments Committee of the University of Sheffield Animal Welfare and Ethical Review Body (AWERB). Work was performed under licence from the UK Home Office and according to recommended standard husbandry conditions [1,2]. The transgenic line used to image the cell membranes in the otic vesicle was *Tg(smad6b:mGFP)*, a gift from Robert Knight [3]. To facilitate imaging, the transgenic line was raised on a *casper* (*mitfa^w2/w2^; mpv17^a9/a9^*) (ZDB-GENO-080326-11) background that lacks all body pigmentation. Embryos were raised in E3 medium (5 mM NaCl, 0.17 mM KCl, 0.33 mM CaCl_2_, 0.33 mM MgSO_4_, 0.0001 % Methylene Blue). Embryonic stages are given as hours post fertilisation (hpf) at 28.5°C. For live imaging, zebrafish were anaesthetised with 0.5 mM Tricaine methylsulfonate and dechorionated.

*Microscopy*

Dechorionated embryos were mounted in 0.8% Low Melting Point Agarose in E3 for microscopy. All imaging was performed at 28°C and using the 488 nm excitation laser line corresponding to the GFP membrane label. Image volume files were cropped to include the structure of interest and a small flanking region of the epithelium surrounding it.

For Airyscan confocal microscopy (Fig 1a), dechorionated embryos were mounted laterally in 0.8% Low Melting Point Agarose in E3 in the centre of a 35mm Wilco glass-bottomed Petri dish. E3 with tricaine was added to the Petri dish after the agarose had set. The image stack of 35 *z*-slices was acquired in a ZEISS LSM 880 Airyscan Confocal Microscope with a 40× objective and a *z*-step size of 1 µm.

Light-sheet microscopy (data for pipeline validation): Dechorionated embryos were mounted in agarose in a glass capillary for imaging on the Zeiss Z1 Light-sheet microscope. The microscope chamber was filled with E3 with tricaine. Image stacks varying from 60 to 110 *z*-slices depending on the age of the embryo were acquired with a 20× objective, 2.3x zoom, and a *z*-step size of 0.5 µm.

*Synthetic data generation*

Synthetic images were generated in MATLAB (2018b, MathWorks) to resemble 3D volumes of folding, cell-membrane-labelled epithelia such as those in the zebrafish otic vesicle depicted in Fig 1a. Each synthetic epithelium was 160 µm x 160 µm along the epithelial plane (XY plane), consisted of about 320 individual cells, and showed two projecting peaks with opposing orientation of folding. The height of the peaks and the curvature of the epithelium were varied to three levels each, such that 9 individual synthetic epithelia were generated (Fig 2a).

The following function defined the surface geometry of each synthetic epithelium generated

$$\begin{aligned} Z= \sqrt{\left| a+X^{2}+Y^{2} \right|} -b\left( \frac{X}{5}-X^{3}-Y^{5} \right)e^{\left( -X^{2}-5Y^{2} \right)}\#\left( S1 \right) \end{aligned}$$

where X and Y are positions on a regular square grid (21 x 21 points) ranging from ‘-4’ to ‘4’ units with an increment of ‘0.4’ units – where each unit = 20 µm. The parameter ‘*a’* influences the radius of curvature of the epithelium (*a* = 5, 20, 80 with a resulting radius of curvature of 106 µm, 134 µm and 200 µm respectively) and ‘*b*’ controls the height of the folded peaks (*b* = 5, 10, 15 with resulting peaks of height 35 µm, 71 µm and 106 µm respectively). Centres of cells (*n* = 320) in the synthetic epithelium were initiated by randomly placing points on this surface, with a minimum distance of 8 µm between them and a padding of 8 µm from the edge of the grid. The resulting set of points were nearly equally spaced.

To convert these surfaces into image volumes, the cell centre positions were then resampled to a volume of isotropic resolution with pixel size of 0.2 µm, resulting in 800 pixels x 800 pixels x >800 pixels (since the *z* dimension was adjusted to accommodate cell positions spanning more than 800 pixels). A Voronoi diagram was generated from the resampled cell centres. The edges of the Voronoi cells were extended 5 µm (26 pixels) orthogonal to the epithelial surface to set cell height and 0.4 µm (2 pixels) in the epithelial plane to set cell membrane thickness. These extended Voronoi edges were used to define a 3D network of polygons as cell membranes. Pixels on the grid that lay within the cell membrane polygons were assigned an intensity value of ‘1’.

The synthetic images generated were then convolved with a Gaussian PSF using a Fast Fourier Transform (FFT)-based convolution (FFT-based convolution; Bruno Luong, MathWorks File Exchange, accessed Oct 2020) to resemble real-world imaging conditions. The PSF was simulated using the PSF Generator plugin in Fiji [4,5], assuming the following experimental parameters: Numerical Aperture of collection objective lens = 0.5, wavelength of illumination = 532 nm, voxel size = 0.2 µm x 0.2 µm x 0.2 µm. The resulting full width at half maximum (FWHM) of the PSF was 0.6 µm x 0.6 µm x 0.8 µm (3 pixels x 3 pixels x 4 pixels). Finally, after combining each of the images (*n* = 9) with the three levels of Gaussian and Poisson noise using the ‘imnoise’ function in MATLAB, 27 synthetic image volumes were generated for performing the validation tests. Ground truth to assess segmentation quality was produced from the 9 uncorrupted image volumes.

Polarity ground truth for the synthetic dataset was generated by producing surface normals to the surface functions described by equation (S1), using the SurfNorm function in MATLAB (version 2018b; MathWorks, Natick MA, US).

*Membrane-based segmentation*

The parameters used to segment our datasets in ACME were different for the synthetic dataset and the real light-sheet data in part, due to differences in voxel resolution (0.2 µm x 0.2 µm x 0.2 µm for the synthetic dataset and 0.1 µm x 0.1 µm x 0.5 µm for the light-sheet data). These parameters were as follows;

For synthetic epithelia:

1. Radius of median filter for denoising – 3.0 pixels (image volumes with noise level 2 and 3), 2.0 pixels (noise level 1)

2. Resampling ratio – 2.5, 2.5, 2.5 (all image volumes)

3. neighbourhood size for membrane signal enhancement filter – 2.0 (noise level 1 and 2), 3.0 (noise level 3)

4. neighbourhood size for Tensor voting – 1.0 (all image volumes)

5. watershed segmentation threshold – 2.0 (noise level 1 and 2), 3.0 (noise level 3)

For fluorescence *in-vivo* data:

1. Radius of median filter for denoising – 0.3 pixels

2. Resampling ratio – 2, 2, 0.39 (resampling to isotropic voxel resolution)

3. neighbourhood size for membrane signal enhancement filter – 0.7

4. neighbourhood size for Tensor voting – 1.0

5. watershed segmentation threshold – 2.0

*Classifying cells*

Cells were classified as lying within the folding structure or the neighbouring epithelium by clustering the centroids of the segmented cells by the mean curvature; that is, the average of the principal curvatures at each vertex [6,7] of the surface mesh generated in the first part of the Origami pipeline (Fig S1). The mean curvature values showed a bimodal distribution, which could be resolved into a population of points on the folding structure and another consisting of points on the neighbouring non-folding epithelium. Cells at the edges of the image volume were discarded to avoid broken cells.


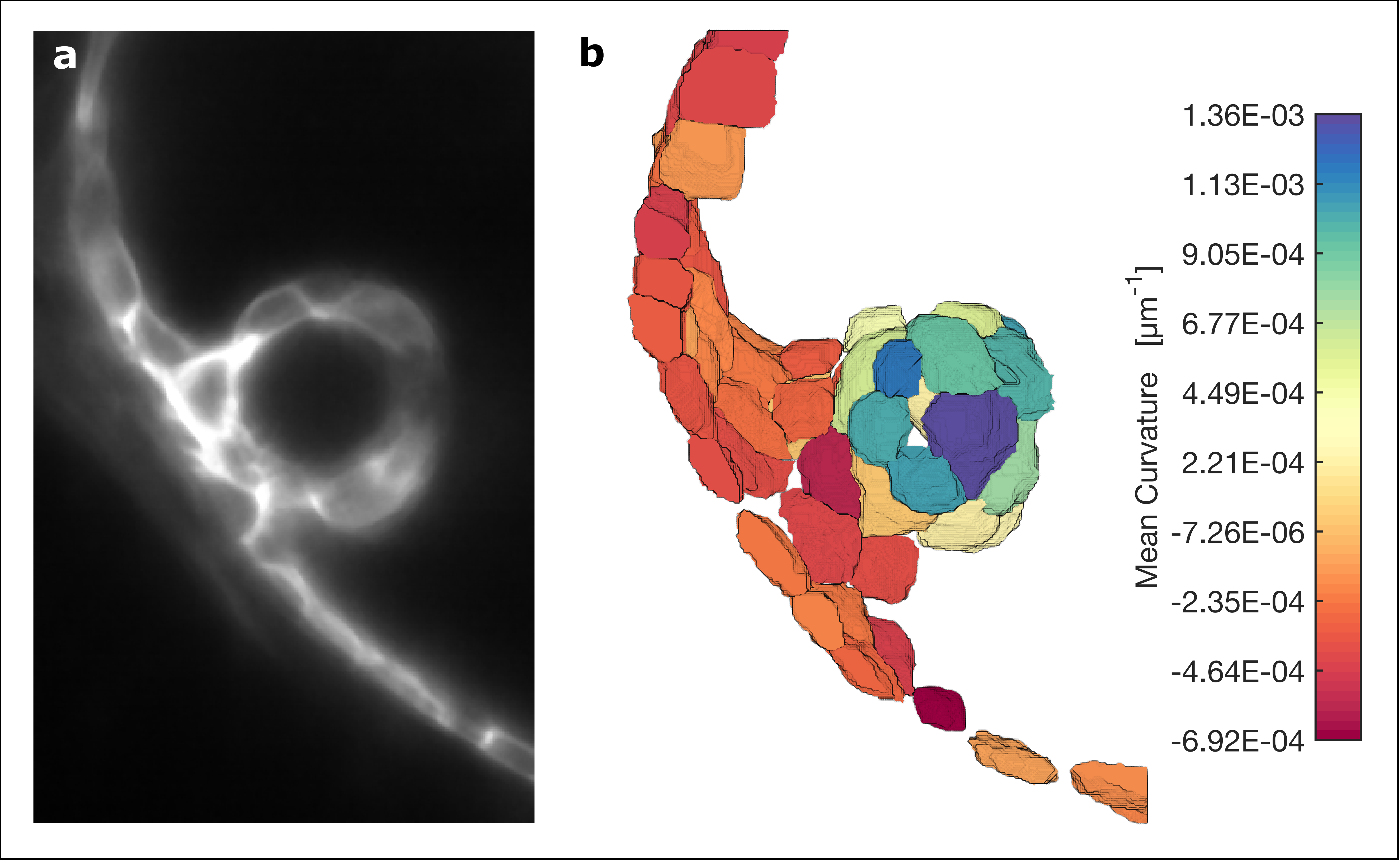


Fig S1: Cell-specific mean curvature of epithelium. a. Single slice through a light-sheet image volume of a region around an anterior projection in the otic vesicle of a 50.5 hpf zebrafish embryo. b. 3D rendering of segmented cells from the same region with individual cells assigned colour values corresponding to the mean curvature at the apical surface of the cell.

# Supplementary References

1. Westerfield M. The Zebrafish Book. A Guide for the Laboratory Use of Zebrafish (Danio rerio), 5th Edition. University of Oregon Press, Eugene (Book). 2007.

2. Aleström P, D’Angelo L, Midtlyng PJ, Schorderet DF, Schulte-Merker S, Sohm F, et al. Zebrafish: Housing and husbandry recommendations. Laboratory Animals. 2020;54: 213–224. doi:10.1177/0023677219869037

3. Baxendale S, Whitfield TT. Methods to study the development, anatomy, and function of the zebrafish inner ear across the life course. Methods in Cell Biology. 2016;134: 165–209. doi:10.1016/bs.mcb.2016.02.007

4. Schindelin J, Arganda-Carreras I, Frise E, Kaynig V, Longair M, Pietzsch T, et al. Fiji: an open-source platform for biological-image analysis. Nature methods. 2012;9: 676–82. doi:10.1038/nmeth.2019

5. Kirshner H, Sage D, Unser M. 3D PSF models for fluorescence microscopy in ImageJ. Proceedings of the Twelfth International Conference on Methods and Applications of Fluorescence Spectroscopy, Imaging and Probes (MAF’11). 2011;1: 154.

6. Rusinkiewicz S. Estimating curvatures and their derivatives on triangle meshes. Proceedings 2nd International Symposium on 3D Data Processing, Visualization and Transmission, 2004 3DPVT 2004. IEEE; 2004. pp. 486–493. doi:10.1109/TDPVT.2004.1335277

7. Shabat Y Ben, Fischer A. Design of porous micro-structures using curvature analysis for additive-manufacturing. Procedia CIRP. Elsevier B.V.; 2015. pp. 279–284. doi:10.1016/j.procir.2015.01.057
