# Supplementary material for: Origami: Single-cell 3D shape dynamics oriented along the apico-basal axis of folding epithelia from fluorescence microscopy data
Source: PLoS Comput Biol. 2021 Nov 1;17(11):e1009063. doi: 10.1371/journal.pcbi.1009063 (PMC8584784; doi:10.1371/journal.pcbi.1009063)
Supplement: Attachment — Submitted filename: Mendonca_Origami_ResponseToReviewers.docx [file pcbi.1009063.s003.docx]

Dear Dr Schneidman

Subject: Response to reviewers’ comments on ‘Origami: Single-cell oriented 3D shape dynamics of folding epithelia from fluorescence microscopy images’ (Manuscript ID: PCOMPBIOL-D-21-00819)

We thank the editor and the reviewers for their time and useful comments. We would like to take this opportunity to emphasise that we believe our manuscript is very well-suited for the remit and readership of PLOS Computational Biology. We think our software ‘Origami’ represents a significant advance in methodology, and as two of the reviewers acknowledged, fills a gap in the current available approaches. Origami is a robust image analysis pipeline that is well characterised with strong metrics for its performance on real biological data and is also presented in an easy-to-use interface that requires no knowledge of MATLAB scripting to run. As an additional valuable resource, we provide a method for generating synthetic folding epithelia which we use for validating Origami.

We have addressed all the reviewers’ comments, reproduced below in red with our responses in-line. Line numbers refer to those in the marked-up version of the manuscript. We hope that our replies and edits to the manuscript clarify the concerns regarding applicability and benchmarking. We appreciate that our use of the term ‘orientation’ needed clarification and have addressed this in our updated manuscript and with a minor change to the Title. Finally, we have deposited our software on GitHub (https://github.com/cistib/origami) for easy public sharing and to enable version control.

Best wishes

Dr Tania Mendonca and co-authors

**Comments to the Authors:**

Reviewer #1: Review of Mendonca et al., “Origami: Single-cell oriented 3D shape dynamics of folding epithelia from fluorescence microscopy images”

In this manuscript, the authors describe new MATLAB-based software to characterize and quantify 3D epithelial cell shape, with only prior knowledge of epithelial orientation. Because many prior approaches require some knowledge of relevant shape features, this problem has not been generally solved. Origami provides a useful step forward in the efforts to capture and quantify meaningful individual cell features within an organized tissue. My comments are mainly minor general questions related to the application of the software to this and other tissues.

• It would be helpful if the authors could comment on different imaging modalities and optimization of imaging parameters. Although segmentation can be carried out prior to analysis using Origami, segmentation seems to be a potential weak link during the Origami validation using synthetic data. Did the authors optimize the imaging for ACME? When using laser scanning confocal microscopy, was the Airyscan modality used? Was it necessary?

We thank the reviewer for their positive and insightful review. To address their minor questions, we would like to clarify that the segmentation method was chosen to suit the data rather than the other way round. The ACME segmentation study (Mosaliganti et al., 2012) reports performance metrics for data from confocal and two-photon microscopy along with synthetic data, whereas our own efforts show good performance with both light-sheet and Airyscan confocal imaging modalities (this manuscript). Although the Airyscan modality provides better resolution data, especially in the axial direction, it does not perform as well as light-sheet microscopy when imaging deeper in the embryo under *in vivo* conditions, so there is a trade-off that does not necessarily result in improved segmentation output from our observations.

Although the performance of Origami is linked to segmentation quality, published benchmarking studies on segmentation software using real-world confocal microscopy data (not Airyscan mode to the best of our knowledge) have consistently reported DICE scores of greater than 80% across the board (Cao et al., 2019; Eschweiler et al., 2019). This gives us confidence that irrespective of segmentation method chosen, errors from Origami would be low (Results, page 11, line 240). Our own validation on light-sheet data show errors in less than 4% of cells analysed (page 12, line 277).

• For the timecourse data, I am not sure if I missed this, but how many embryos were used for each timepoint?

We analysed data from three different embryos at each time point. We now clarify this on page 14, line 306.

• Did the authors test how consistent the data are between wild type embryos at any one individual timepoint? This might help to establish the kind of noise one might expected in the data, as cells and embryos are not exactly alike.

We do expect variation between wild-type embryos at individual timepoints, however, we did not have the statistical power (*n*=3) to make inferences on this variation. Ultimately, the aim of our validation experiment was to analyse developmental timepoints that were disparate enough to display distinct cell shape dynamics and our results show that we could successfully distinguish between the structures with opposing shape signatures at these timepoints.

Reviewer #3: This manuscripts present a MATLAB program to find the apical-basal axis of each cell in an arbitrarily curved epithelia, and then calculates cell-based shape metrics based on this axis. Shape information of a cell with respect to its apical-basal axis is an essential first step in trying to understand and model many aspects of epithelial morphogenesis. There is some existing work in the literature but not a generally useful approach so the current approach fills an important gap. The chosen method is straightforward and good (fits a surface mesh to the centroids of all cells and then calculates surface normals for all faces after handling mesh defects). The manuscript is generally clearly written and explains the approach at the right level of detail. The method is nicely validated using appropriate metrics on synthetic data and real data from the zebrafish inner ear.

I had a few minor concerns:
Abstract/title should be more specific as to what “orientation” means- apical-basal, planar cell polarity, major axis of a cell are all possible interpretations of “orientation”. Should include “apical-basal” in abstract and maybe title to clearly indicate what the point of the paper is.

We are pleased that the reviewer agrees that our work is an important contribution to the field and are thankful for their supportive comments. We accept that our use of the term ‘orientation’ has caused some confusion, and have edited the manuscript extensively – particularly the Abstract and Introduction to help improve clarity. We now specify apico-basal orientation in the Abstract (page 2, line 32) and Title as suggested by the reviewer.

Calculation of moments. It appears the authors are using the vertex positions of the surface mesh to calculate a cell’s moments. This could run into errors if the mesh is not regular (e.g. smaller faces/more vertices in one part than another. There is also the issue of calculating moments of the surface shell rather the whole volume which would give unexpected results on cells with thin processes such as neurons or pseudostratified epithelia. I think it would be better to convert the mesh into an image—i.e. fill it with isometric voxels and then compute the moments using the set of voxels.

Origami calculates geometric moments using the algorithm presented in (Pozo et al., 2010). This algorithm uses as input a triangular mesh representing the surface of the object. The formula only requires the vertex positions and their connectivities. However, the algorithm computes the exact moments defined by the corresponding continuous integral in the object interior. The method is very robust to variations in the mesh size and quality, as demonstrated in (Pozo et al., 2010). If this exact mesh-based method were not available, we agree with the reviewer that the moments should be computed by the suggested image-based method. We recognise that the original description in our manuscript could lead to this type of misunderstanding. We have now edited the manuscript to convey our method more clearly – see page 5, line 113-115 and page 7, line 168-179.

Reviewer #2: The manuscript presents Origami, a microscopy image analysis pipeline devoted to the quantification of shape in oriented biological structures. Origami relies on a mesh representation of segmented objects, and then computes shape features from the 3D geometrical moments introduced by a subset of the authors in a 2011 IEEE PAMI paper.

I have major concerns about three aspects of this work:

1) Novelty: the technical/methodological/algorithmic novelty of Origami is not significant and in my opinion too minor to fit the scope of PLOS CompBio. Providing a freely-available MATLAB implementation of a compilation of a bunch of existing methods (triangulation and meshing, mesh cleaning/processing, geometrical moments) is definitely useful but of limited computational innovation. Although there doesn't seem to be any, if novel computational methods had to be developed for this pipeline (as opposed to assembling existing ones), the authors should highlight them more clearly.

We thank the reviewer for their detailed comments. We appreciate their concerns, but disagree that our contribution is not significant. The consolidation of tools into a well-characterised, easy-to-use pipeline is an innovation of itself and is of great value to the biological research community. We feel that a requirement for algorithmic novelty simply pushes for needlessly over-engineered solutions. We hope our answers to the reviewer’s second and third questions below on significance and benchmarking will clarify the premise and novelty of our work and help alleviate their concerns.

2) Significance: although Origami is described as a way to quantify cell shape features in general, it entirely relies on prior knowledge of the epithelium organisation. As such, it can only provide oriented shape features when orientation is known a priori, which makes it limited to very specific settings. The introduction is particularly confusing as it emphasizes the issue of orienting cells along biologically relevant axis, which *is precisely not* solved by Origami. Origami seems to focus mostly on implementing a new method to extracts shape features along a known polarity axis, which can per se be valuable but it not spelled out clearly here. The authors should work on a major rewrite to a) define the scope of their work better and b) outline which problem Origami is aiming to solv, and c) clarify what Origami can bring over other approaches (see 3)).

We have now made edits to our manuscript throughout, especially concerning our use of the words ‘orientation’ and ‘direction’, with respective different meanings. We hope that this has improved their clarity.

Origami computes a *direction vector* for each cell in the epithelium, regardless of specimen geometry, developmental stage and complexity of folding, whereas the few other software packages available that provide similar functionality assume specific geometries or cell-shape features. While the direction vector can shift continuously having any value in a sphere, its *orientation* can only have two values (difference only in sign): either it has the correct orientation or it should point in the opposite one. Origami automatically extracts the apico-basal direction vector. It is only the orientation that may need additional information or manual revision (flipping).

We recognize that there is no clear standard for the definition of the terms ‘orientation’ and ‘direction’, whose meaning are area-dependent, and even language-dependent. We believe that this ambiguity induced the reviewer to misinterpret our statement that the epithelial apico-basal *orientation* must be known, as a requirement to know its *direction vector*. To make it clear, the direction vector is automatically computed by Origami with no additional information. Knowing the epithelial apico-basal organisation helps us make the decision of which orientation (of the two opposite options) the vector should face to point towards the apical surface. We have now made changes to the manuscript on page 6, lines 145-151, to explain this and make the definition unambiguous.

3) Comparison/benchmarking: the authors dismiss several related methods on the basis that they are "specific to the geometry of the specimen"/"applicable only if a shape feature is known". I do not think this argument is valid since the whole orientation/polarity estimation of Origami relies precisely on the known geometry/features of the considered epithelium. Comparative experiments must be carried out to quantify how much the shape features extracted by Origami bring over those extracted eg by LongAxis. A sensible experiment could for instance be to orient the "long axis" of the cells along the known apico-basal axis, and compare the robustness of the features extracted by LongAxis and Origami. An additional important aspect to investigate is the relevance of the mesh representation. How would the skewness, transversal/longitudinal spread, sphericity and surface area features of a simple ellipse fitted on the voxel data of the segmented cell such that its major axis is aligned to the known apico-basal axis fare against the geometrical moments proposed here?

Origami provides a generalised solution to computing directional vectors along the epithelial apico-basal axis. We hope our previous answer sheds some light on this argument.

The LongAxis software (Carney et al., 2020) computes the 'long axis’ of the cell using an ellipsoid fit. In their case (cells of the optic cup), this long axis represents an axis of interest, but the long axis of a cell may not always fall along a biologically relevant axis. Additionally, the long axis of the cell can change over time and space during morphogenetic epithelial folding events, for example where squamous cells in an epithelial sheet can change to acquire a columnar morphology (Pearl et al., 2017) or vice versa. The orientation of the long axis vector is set in ‘LongAxis’ by computing the convergent point of the cells in the curved optic cup; such a convergent point may not exist in epithelia where the cells are squamous in shape, or in open structures that don’t curve in on themselves. This is illustrated in the figure below where the vectors generated from LongAxis (red) fall arbitrarily within the plane of the epithelium and do not represent the apico-basal axis (blue arrows generated using Origami).

Figure: Comparison of cell polarity vectors generated by LongAxis (in red) and vectors generated by Origami (blue arrows).

What the reviewer suggests as a sensible comparison is to contrast cell shape features derived from LongAxis to those from Origami. LongAxis outputs cell length, width and length/width ratio by applying an ellipsoid fit, and volume by summing the voxels enclosed by each segmented cell. Origami computes comparable features using geometric moments. A key additional shape feature that Origami provides is ‘Skewness’ which captures asymmetry in cell mass distribution along the apico-basal axis (page 8) and is computed as the third central moment of the volume enclosed by the segmented cell. Positive skewness suggests apical cell constriction and/or basal relaxation while negative skewness could indicate apical relaxation and/or basal constriction, making skewness a valuable metric that provides insight into the mechanisms underlying epithelial folding when combined with biomechanical and genetic manipulations. In contrast, if the cell shape were represented by a fitted ellipsoid, all skewness information would be removed: an ellipsoid is symmetric by definition, so that its skewness is always null. We could calculate direction-variant cell moments by referencing vectors generated using a different method to Origami – such as the long axis of an ellipsoid fit, assuming such an axis lies along an axis of interest – but there is no computational advantage to doing this (time to compute vectors for the segmented cells in the Figure above – from Origami: ~20ms; from the ‘ellipsoid_fit’ function in LongAxis: ~80ms, on a machine with 16GB RAM and a 2.8GHz processor).

On a more minor note, I would encourage the authors to make their code available on a github repository as this would facilitate traceability and reuse.

We have now made our code publicly available on GitHub (https://github.com/cistib/origami).

References:

Mosaliganti KR, Noche RR, Xiong F, Swinburne IA, Megason SG. ACME: Automated Cell Morphology Extractor for Comprehensive Reconstruction of Cell Membranes. Murphy RF, editor. PLoS Comput Biol. 2012;8: e1002780. doi:10.1371/journal.pcbi.1002780

Cao J, Wong MK, Zhao Z, Yan H. 3DMMS: Robust 3D Membrane Morphological Segmentation of C. elegans embryo. BMC Bioinformatics. 2019;20: 176. doi:10.1186/s12859-019-2720-x

Eschweiler D, Spina T V., Choudhury RC, Meyerowitz E, Cunha A, Stegmaier J. CNN-based preprocessing to optimize watershed-based cell segmentation in 3d confocal microscopy images. Proceedings - International Symposium on Biomedical Imaging. IEEE Computer Society; 2019. pp. 223–227.

Pozo JM, Villa-Uriol M-C, Frangi AF. Efficient 3D Geometric and Zernike Moments Computation from Unstructured Surface Meshes. IEEE Trans Pattern Anal Mach Intell. 2011;33: 471–484. doi:10.1109/TPAMI.2010.139

Carney KR, Bryan CD, Gordon HB, Kwan KM. LongAxis: A MATLAB-based program for 3D quantitative analysis of epithelial cell shape and orientation. Dev Biol. 2020;458: 1–11. doi:10.1016/j.ydbio.2019.09.016

Pearl EJ, Li J, Green JBA. Cellular systems for epithelial invagination. Philos Trans R Soc B Biol Sci. 2017;372: 20150526. doi:10.1098/rstb.2015.0526
